# Sleep in Farm Adolescents

**DOI:** 10.1111/jrh.12341

**Published:** 2018-11-28

**Authors:** Ian Janssen, Richard L. Berg, Barbara Marlenga, William Pickett

**Affiliations:** ^1^ School of Kinesiology and Health Studies Queen's University Kingston Ontario Canada; ^2^ Department of Public Health Sciences Queen's University Kingston Ontario Canada; ^3^ Biomedical Informatics Research Center Marshfield Clinic Research Institute Marshfield Wisconsin; ^4^ National Children's Center for Rural and Agricultural Health and Safety, National Farm Medicine Center Marshfield Clinic Research Institute Marshfield Wisconsin

**Keywords:** adolescent, epidemiology, farmers, health surveys, sleep

## Abstract

**Purpose:**

The objectives were to: (1) describe sleep timing and patterns among adolescents who live or work on farms; (2) compare these sleep characteristics to those of nonfarm adolescents; (3) explore whether the above sleep and farm versus nonfarm differences varied by age and gender.

**Methods:**

Participants were aged 11‐16 years and were abstracted from the 2014 Canadian Health Behaviour in School‐aged Children study. Records from 2,160 farm adolescents were frequency matched (by school, gender, and grade) to records from 2,210 nonfarm adolescents. Participants self‐reported their bedtimes and wake‐up times on weekdays and weekends.

**Findings:**

Among farm adolescents, average nightly sleep duration (hours:minutes) ranged from 08:34 among 14‐ to 16‐year‐old girls to 09:21 among 11‐ to 13‐year‐old girls. Approximately 24% to 32% of farm adolescents did not meet minimal sleep duration targets. For 11‐ to 13‐year‐olds, sleep characteristics did not differ according to farm status. However, for 14‐ to 16‐year‐olds, farm adolescents had shorter sleep durations than nonfarm adolescents (23 minutes for boys, *P* = .02; 20 minutes for girls, *P* = .06). Furthermore, a greater proportion of 14‐ to 16‐year‐old farm boys had sleep duration values less than the recommended 8 hours/night (27.7% vs 19.6%, *P* = .05).

**Conclusions:**

This study profiles the sleep experiences of 11‐ to 16‐year‐old farm adolescents. Almost 1 in 3 of these adolescents did not get adequate sleep. Older adolescents who lived or worked on a farm slept less than comparably aged nonfarm adolescents. This may reflect their participation in morning chores essential to the farm operation and may increase their injury risk.

Sleep deprivation has emerged as a leading health problem among adolescent populations.^1^ Adolescence represents a key developmental period in terms of physical and emotional health,^2^ and insufficient sleep may have effects that are detrimental to the health of young people. Such effects include elevated risk factors for chronic disease (eg, obesity, insulin resistance),^3^, ^4^ effects on the brain (eg, decreased cognition, memory, and attention),^4^, ^5^ and increased risk for physical injury.^6^

Adolescents who live or work on farms may be especially vulnerable to insufficient sleep. Many of these young people need to get up early every morning in order to perform work that is essential to the farm operation.^7^ Some of this work involves operation of machinery, working with large animals, and working at heights, each of which has inherent risks for injury.^8^ When insufficient sleep is combined with these work requirements, concerns for injury are particularly relevant.

Public heath guidelines for sleep duration have been established for pediatric populations.^9^, ^10^ These guidelines recommend that 5‐ to 13‐year‐olds get 9‐11 hours of sleep each night and that 14‐ to 17‐year‐olds get 8‐10 hours of sleep. A growing body of evidence indicates that large portions of the general adolescent population are not getting sufficient sleep.^1^, ^11^, ^12^ For instance, 30% of 10‐ to 17‐year‐old Canadian children and adolescents do not get enough sleep to meet minimal sleep duration targets.^11^ However, little is known about young people from farm backgrounds. Another phenomenon observed in general adolescent populations is weekend “catch‐up” sleep, where sleep debt compiled during the school week is in part addressed with longer sleep durations on the weekend.^13^, ^14^ Whether or not catch‐up sleep occurs within farm populations remains unknown, and ongoing surveillance is hence warranted. Given the gendered and age patterns of work observed on farms,^15^ this phenomenon may be particularly important among older boys.

The Canadian Health Behaviour in School‐aged Children (HBSC) study provides an opportunity to explore these questions in a robust, national sample of young people given that this study included items that assess sleep characteristics^11^, ^16^ and identify adolescents aged 11‐16 years living or working on farms.^17^, ^18^ Our hope was that an epidemiological analysis of the Canadian HBSC study would provide foundational evidence for health promotion strategies aimed at adolescents on farms.

## Methods

### Study Design and Sample

This study was based on data from cycle 7 of the Canadian HBSC study.^19^ Data were collected in 2014 from grade 6‐10 students in all provinces and territories. To ensure representativeness, the sample was stratified by province/territory, type of school board (public vs separate), urban‐rural geography, school population size, and language of instruction (French or English). Adolescents from private or home schools, schools on First Nation or Inuit reserves, and street youth were not included; together these adolescents represent ∼7% of youth in the target age.^20^ Explicit or implicit consent, depending on school board requirements, was obtained from school administrators, parents, and participating students. Response rates were 77% at the student level. Ethics clearance was obtained from ethics boards at Queen's University, Health Canada, and the Public Health Agency of Canada.

The primary goal of the present study was to describe sleep characteristics of adolescents from farm settings, and to compare those to adolescents from nonfarm settings. Participants who indicated that they lived or worked on a farm formed the farm group, and all other participants were eligible for the nonfarm comparison group. Inclusions were as follows: (1) participated in the HBSC and provided complete information on all key study variables, and (2) aged 11‐16 years and in grades 6‐10. There were gender, age, and geographical differences between farm and nonfarm participants, which indicated that farm students were more likely to be older, male, and from rural areas. To ensure that our comparisons reflected effects of the farm environment with minimal confounding by regional differences and other factors, records for farm youth were frequency matched, in a 1:1 ratio, with records from nonfarm youth from the same school, by grade, age, and gender.

**Figure 1:**
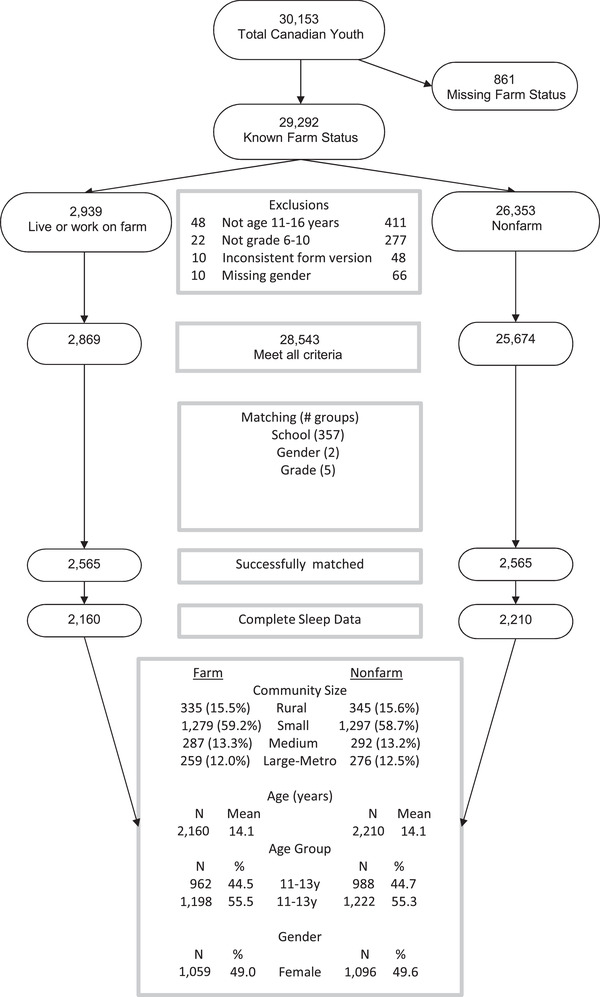
Demographic Description of Samples of Farm and Nonfarm Youth, Including Matching Criteria.

The full 2014 Canadian HBSC sample consisted of 30,153 participants from 377 schools, and 29,292 of these participants reported their farm status (Figure 1). From these, we identified 2,948 farm youth and 26,316 nonfarm youth. Following exclusions for the matching criteria, we identified 2,869 farm youth and 25,674 nonfarm youth from 357 schools. We successfully matched 2,565 of each group in a 1:1 ratio. In the matched analysis cohort used for the final analyses, complete information on sleep was available in 2,160 farm and 2,210 nonfarm participants.

#### Demographic Information

Participating adolescents reported whether they lived or worked on a farm (yes or no), their gender (male or female), and month and year of birth. The later information coupled with the date of survey administration were used to estimate age in years. Two age groups were formed: 11‐ to 13‐year‐olds and 14‐ to 16‐year‐olds. Age was categorized in this manner because, as explained below, there is a change in the sleep duration recommendation from 13 to 14 years of age.^9^, ^10^

#### Sleep

Participants were asked to report, for the past week, the typical time they turned out the lights to go to sleep and the typical time they woke up in the morning (hours:minutes), separately for weekdays and weekends. From this information we calculated sleep durations for weekday nights and weekend nights, and averages across all 7 nights of the week. Weekend catch‐up sleep was calculated by subtracting weekday sleep duration from weekend sleep duration.^13^, ^14^ We also determined if participants’ average sleep duration was shorter than the recommended 9‐11 hours per night for 11‐ to 13‐year‐olds and 8‐10 hours per night for 14‐ to 16‐year‐olds.^9^, ^10^

### Statistical Analysis

For data analyses we used SAS 9.4 (SAS Institute Inc., Cary, North Carolina). Descriptive summaries characterized the final samples of farm and nonfarm adolescents by age, sex, and other features. Procedures for analysis of complex survey data (eg, PROC SURVEYREG) were used in the primary analyses to assess the associations of farm status with sleep times and durations. All analyses were adjusted for the weighting and clustering of the complex HBSC sampling design. Results were deemed statistically significant at the nominal 5% level (*P* <.05) without adjustment for multiple comparisons.

## Results

The sociodemographic characteristics of the matched farm and nonfarm adolescents are shown in Figure 1. Farm and nonfarm adolescents were almost perfectly balanced on community size, age, and gender. The race and self‐reported socioeconomic status were also comparable between farm and nonfarm adolescents.

Mean values for the sleep timing, sleep duration, and weekend catch‐up sleep variables are described in Table 1. On weekdays, the only significant differences between farm and nonfarm adolescents occurred within adolescents aged 14‐16 years. Specifically, on average, girls who lived or worked on farms went to bed 11 minutes earlier than nonfarm girls (*P* = .047) and woke up 8 minutes earlier (*P* = .053), while farm boys went to bed 1 minute later than nonfarm boys (*P* = .86) and woke up 10 minutes earlier than nonfarm boys (*P* = .041). There was no significant difference in sleep duration between girls and boys. On weekends, there were no differences in bedtimes, wake‐up times, or sleep durations for boys and girls aged 11‐13 years. However, 14‐ to 16‐year‐old boys and girls who lived or worked on a farm woke up earlier (29 minutes for boys, *P* = .007; 28 minutes for girls, *P* = .002) and had shorter nightly sleep durations (23 minutes for boys, *P* = .019; 20 minutes for girls, *P* = .064) than 14‐ to 16‐year‐olds who did not live or work on a farm. Furthermore, weekend catch‐up sleep was 23 minutes shorter (*P* = .035) in 14‐ to 16‐year‐old farm girls. When averaged across all 7 nights of the week, the only significant difference in sleep duration occurred for 14‐ to 16‐year‐old boys; farm adolescents slept 14 min/night less (*P* = .026).

**Table 1:** Mean Bedtimes, Wake‐Up Times, Sleep Durations, and Weekend Catch‐Up Sleep Within Farm and Nonfarm Youth According to Age and Gender

	Averaged Across All Days of the Week				Weekdays			Weekends		
Farm, Age, Gender Group	Bedtime	Wake‐Up Time	Sleep Duration	Bedtime	Wake‐Up Time	Sleep Duration	Bedtime	Wake‐Up Time	Sleep Duration	Weekend Catch‐Up Sleep
*11‐ 13‐year‐old boys*										
Farm	22:07 (00:05)	07:34 (00:03)	09:26 (00:04)	21:41 (00:05)	06:59 (00:03)	09:18 (00:05)	23:13 (00:08)	09:02 (00:07)	09:48 (00:06)	00:30 (00:06)
Nonfarm	22:10 (00:06)	07:36 (00:03)	09:25 (00:05)	21:45 (00:05)	07:00 (00:03)	09:15 (00:05)	23:15 (00:08)	09:08 (00:06)	09:53 (00:07)	00:38 (00:06)
*11‐ 13‐year‐old girls*										
Farm	22:08 (00:04)	07:30 (00:03)	09:21 (00:04)	21:48 (00:04)	06:53 (00:03)	09:04 (00:04)	22:58 (00:05)	09:02 (00:06)	10:03 (00:06)	00:58 (00:07)
Nonfarm	22:12 (00:03)	07:34 (00:03)	09:22 (00:04)	21:48 (00:03)	06:56 (00:03)	09:07 (00:04)	23:10 (00:05)	09:10 (00:06)	09:59 (00:07)	00:52 (00:06)
*14‐ 16‐year‐old boys*										
Farm	23:01 (00:05)	07:37 (00:04)	08:36 (00:05)	22:35 (00:04)	06:56 (00:04)	08:20 (00:06)	00:04 (00:07)	09:21 (00:08)	09:16 (00:07)	00:56 (00:07)
Nonfarm	23:02 (00:05)	07:53 (00:03)*	08:50 (00:04)*	22:34 (00:04)	07:06 (00:03)*	08:31 (00:05)	00:11 (00:08)	09:50 (00:07)*	09:39 (00:05)*	01:07 (00:05)
*14‐ 16‐year‐old girls*										
Farm	22:52 (00:04)	07:26 (00:03)	08:34 (00:05)	22:28 (00:04)	06:43 (00:03)	08:15 (00:05)	23:52 (00:07)	09:13 (00:07)	09:20 (00:07)	01:05 (00:06)
Nonfarm	23:02 (00:04)	07:39 (00:03)*	08:36 (00:03)	22:39 (00:04)*	06:51 (00:03)	08:11 (00:04)	00:01 (00:06)	09:41 (00:07)*	09:40 (00:08)	01:28 (00:08)*

Data presented as mean in hr:min (standard error in minutes). Bedtimes and wake‐up times are based on a 24‐hour time format.

^*^Significantly different from estimate for farm youth within same age and gender group.

**Figure 2:**
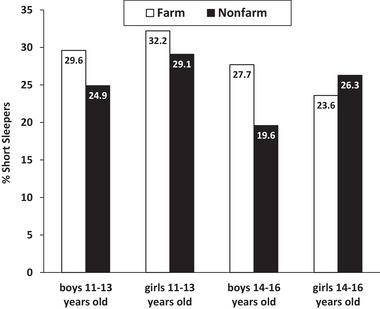
Proportion (%) of Farm and Nonfarm Youth Who Slept Less Than the Recommended Duration, When Averaged Across All 7 Days of the Week, According to Age and Gender.

Figure 2 illustrates the proportion of farm and nonfarm youth who slept less than the recommended duration (ie, <9 hours/night for 11‐ to 13‐year‐olds, <8 hours/night for 14‐ to 16‐year‐olds), when averaged across all 7 nights of the week, according to age and gender. The largest difference occurred within 14‐ to 16‐year‐old boys; a much greater proportion of farm adolescents slept less than the recommended duration (27.7% farm, 19.6% nonfarm, *P* = .051).

## Discussion

This study profiles the sleep experiences of 11‐ to 16‐year‐olds who live or work on a farm. Almost 1 in every 3 of these adolescents did not get adequate sleep. Older farm adolescents, but not younger farm adolescents, slept less than comparably aged nonfarm adolescents, which in part reflected earlier wake‐up times and less weekend catch‐up sleep. Of particular concern is the observation that a greater proportion of 14‐ to 16‐year‐old boys who lived or worked on farms did not get adequate sleep compared to 14‐ to 16‐year‐old nonfarm boys.

Previous analyses based on the full 2014 Canadian HBSC study sample indicated that the typical grade 6‐10 student sleeps approximately 9 hours/night, when averaged across all 7 nights of the week, and that 28% sleep less than the recommended amount.^11^ That study, and others, also revealed that boys’ and girls’ sleep durations and patterns are similar, but that adolescents go to bed later and sleep less as they get older and attempt to address the weekday sleep debt by sleeping more on the weekend.^11^, ^21^, ^22^

Chaput et al have argued that sleep should be taken more seriously by public health and clinical audiences.^23^ Our findings certainly support the notion that insufficient sleep is a common problem among farm adolescents and that sleep should be taken seriously by people interested in the health of rural and agricultural populations. The inherent physical risks associated with farm work,^8^ when coupled with a heightened injury risk attributable to sleep debt,^6^ means that adolescents who live or work on farms are very vulnerable to injury. It is our hope that these findings could lead to health promotion initiatives aimed at stressing the benefits of sleep in farm communities. If successful, such initiatives could reduce injury rates given the dangerous nature of farm work^8^ and the culture and tradition of risk‐taking lifestyles among farm adolescents.^17^, ^18^ However, we need evidence from randomized controlled trials that would support specific intervention strategies.

There are notable strengths of this study. The findings are based on a robust and contemporary dataset. The analysis of sleep data in farm adolescents is novel and applies findings to new evidence‐based recommendations on sleep duration. As with any study, this study was not void of limitations. Although self‐reported and objective sleep measures are well correlated,^24^ the self‐reported nature of the data undoubtedly led to some misclassification of the sleep variables. The HBSC study is not designed to be representative of farm populations. Furthermore, the manner in which farm adolescents were identified on the questionnaire did not allow us to specifically identify adolescents who were performing farm work, or different kinds of farm work (eg, work with livestock, work with machinery), which is important from an injury prevention standpoint.

## Conclusions

This study provides contemporary data on the sleep behaviors of adolescents who live and work on farms. Insufficient sleep was a common phenomenon in this population. Older farm adolescents, and boys in particular, slept less than their nonfarm peers. This may reflect their participation in the morning chores that are essential to the farm operation and may increase their risk of injury.
